# Constraints are the solution, not the problem

**DOI:** 10.3389/fnhum.2014.00324

**Published:** 2014-05-20

**Authors:** Sebastian Wallot, Damian Kelty-Stephen

**Affiliations:** ^1^Department for Culture and Society, Interacting Minds Centre, Aarhus UniversityAarhus, Denmark; ^2^Department of Psychology, Grinnell CollegeGrinnell, IA, USA

**Keywords:** eye-movement fluctuations, reading, fixations, saccades, scale invariance, ultrafast reading times

Reichle and Reingold (2013) presented the hypothesis that parafoveal preview is a requirement for the average fixation during reading in order for lexical processing to control eye movements. Taking 240 ms as the customary fixation duration in reading a single word, they arrive at their conclusion by decomposing these 240 ms into stages of a strictly serial-processing model. This subtractive accounting finds the visual system coming up short so long as lexical processing is strictly foveal, that is, so long as lexical processing addresses only the word directly in front of the fovea during a fixation. Neurophysiological estimates of latencies in retinal transmission to the brain and in visual encoding leave little more than 60 ms for lexical processing and 90 ms for programming the next saccade. Reichle and Reingold's solution to this processing bottleneck is to propose that lexical processing of the next word *usually* begins parafoveally, that is, during the fixation of the current word.

We question the generality of the solution that Reichle and Reingold (2013) offer. Rather the observation of comparatively short fixation durations may tie in with a wider range of findings on remarkably fast response times in which cognitive, neural, and physiological events unfolding at various time scales conspire to poise the human organism for a variety of stimuli and dramatically reduce the time needed to arrive at sensible response from stimulus onset (Wallot and Van Orden, 2012). We argue that the solution to the problem can be found by investigating the contextual constraints organizing cognitive activity.

## Paravfoveal processing

The requirement of parafoveal processing to make this serial temporal accounting work raises as many questions as it may resolve. For instance, word skipping rates (counting out refixations) during reading vary between 20% and 50% (Starr and Rayner, 2001; Demberg and Keller, 2008), suggesting that parafoveal preview will often not precede foveal fixation, leaving later fixations to resolve the processing bottleneck all over again. Also, the proposed role for parafoveal fixation suggests that the very first fixation during reading must be on average longer than the subsequent fixations, as no parafoveal preview is possible. However, this is not borne out in empirical data (e.g., Rayner, 1977).

It is noteworthy that the temporal accounting behind proposals for parafoveal fixations depend heavily on the centrality of local lexical features for text reading (Rayner and Reichle, 2010). However, the availability of general invariants on the sub-lexical, lexical, or syntactic scale of reading are in doubt (see Frost, 2012). Complicating matters further, local lexical features (such as word frequency) play no substantial role in connected text reading (Wallot et al., 2013). Hence, insofar as one considers text reading as the target phenomenon that theories of reading should explain, it is questionable whether local lexical features are really at the core of naturalistic reading.

## Saccades, fixations, and power-law scale invariance

**Figure 1:**
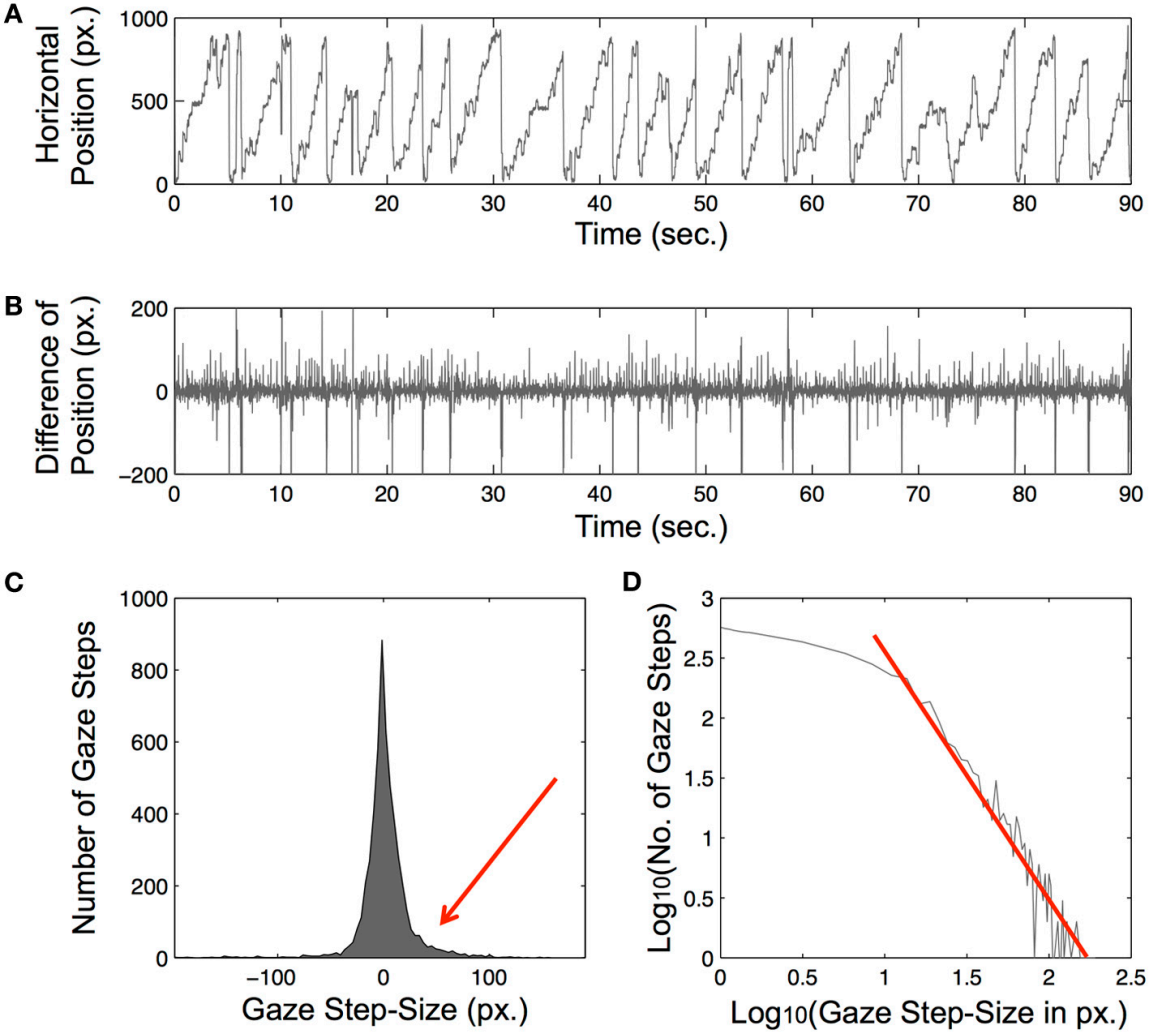
**Scaling in eye-movement fluctuations**. **(A)** Record of horizontal eye-gaze positions in pixels during text reading. The data were recorded using an ASL D6 eye-tracker (sampling rate: 60 Hz; resolution: 0.25°), with 1 pixel ≈0.02°. **(B)** Difference of position-record (Gaze step-size) from **(A)**. In this record, periods of large fluctuations indicate saccadic movements, and periods of small fluctuations indicate fixations. **(C)** Histogram of gaze steps from **(C)**. **(D)** Log-log plot of the right side of Gaze step-size distribution. The logarithm of the Gaze step-size falls linearly off as a function of the logarithm of the number of Gaze steps. However, notice the deviation form linearity on the graph for the smallest step-sizes. This deviation occurs at around a step-size of 10 px., and might be the results of aliasing effects due to the minimal spatial resolution of the eye-tracking system.

Reliable algorithmic classifications of eye movements along a fault line between saccades and fixations has been elusive (Karsh and Breitenbach, 1983) and algorithmic differences in fixation identification lead to diverging results (Salvucci and Goldberg, 2000). When examined strictly in terms of distances between consecutive samples of an eye-tracking device, the distribution of the raw eye-movement record conforms to a unimodal power-law-like distribution. Hence, fixations and saccades do not reveal themselves in terms of a bi-modal (or multimodal, for the case of microsaccades; e.g., Rolfs, 2009) distribution. The scale-invariant, power-law-like form suggests that eye-movement fluctuations are a scale-free process (Stephen and Mirman, 2010; Coey et al., 2012), where the smallest fluctuations (i.e., the eye's tremor during a fixation) blend seamlessly with the largest fluctuations (i.e., long saccadic movements; see Figure 1). The scale-invariance in eye-movement fluctuations only exacerbates the algorithmic challenge of identifying a context-general threshold for distinguishing fixational from saccadic fluctuations in behavioral eye-tracking data.

## Constraints as solution?

The dominant interpretation of power law relationships in empirical data is that they reflect subtle coordination of very many nested constraints, driven by the slowest time-scale constraints and cascading downwards into progressively faster bodily and neuro-cognitive events, each constraining and shaping the next (Van Orden et al., 2003; Ihlen and Vereijken, 2010). For the case of eye-movements during reading, scale invariance implicates factors on broader time-scales than those for individual-word lexical processing, such as reader expertise and the text's emerging meaning (Wallot et al., 2013), or the experimental protocol (Van Orden et al., 2010). Constraints delimit the amount of necessary information processing by setting up the organization of the neuro-cognitive system in a way that only relevant information needs to be processed as opposed to whole possible informational content of a stimulus (van Rooij, 2012; Wallot and Van Orden, 2012). The serial model requiring parafoveal preview appears to imply that each word is an entirely new obstacle to be reckoned with as though the neuro-cognitive system had not already processed hundreds of words leading up to it.

The problem that fast reading times pose might not arise so much from neurophysiological limits but from the premise that only short-range factors (i.e., lexical word features) lead serially to short-range effects (i.e., fixation durations). Scale invariance in eye-movements (Stephen and Mirman, 2010; Coey et al., 2012; Kelty-Stephen and Mirman, 2013) and written language (Montemurro and Pury, 2002) motivates a re-conceptualization of the reading process in terms of cascade formalisms. Cascades constitute a class of formalisms characterizing random processes as the hierarchical spreading or clustering of events nested across very many scales (e.g., words nested within sentences within paragraphs within passages; Turcotte et al., 2002). Importantly, they manifest in the scale-invariant properties indicative of nonlinear correlations across space or time. Such cascade-driven nonlinear dependence across time entail that fixation and saccade are not, as Reichle and Reingold (2013) suggest, chained together as serially independent events. Parafoveal preview resolves a paradox inherited from the purely serial model of reading. Cascade-based models of reading might allow for fast reading times exactly by taking at face value the possibility that our perceptual systems allow us to read through coordination of sub-processes across different time-scales and across words, sentences, or texts.

### Conflict of interest statement

The authors declare that the research was conducted in the absence of any commercial or financial relationships that could be construed as a potential conflict of interest.
